# Knowledge, Attitudes, and Practice Patterns of Lung Cancer Screening Among Physicians in Saudi Arabia

**DOI:** 10.7759/cureus.51842

**Published:** 2024-01-08

**Authors:** Rayan A Qutob, Ibrahim Ali Almehaidib, Sarah Saad Alzahrani, Sara Mohammed Alabdulkarim, Haifa Abdulrahman Abuhemid, Reema Abdulrahman Alassaf, Abdullah Alaryni, Abdullah Alghamdi, Eysa Alsolamy, Abdullah Bukhari, Abdulwahed Abdulaziz Alotay, Mohammad A Alhajery, Abdulrahman Alanazi, Fahad Ali Faqihi, Mohanad Khalid Almaimani

**Affiliations:** 1 Department of Internal Medicine, College of Medicine, Imam Mohammad Ibn Saud Islamic University, Riyadh, SAU; 2 Department of Internal Medicine and Adult Critical Care Medicine, Dr. Sulaiman Al Habib Medical Group Holding Company, Riyadh, SAU; 3 Department of Medicine, University of Jeddah, Jeddah, SAU

**Keywords:** physicians, lung cancer, practice, attitudes, knowledge

## Abstract

Background: Lung cancer remains the primary cause of death connected to cancer on a worldwide scale. Obtaining a deep understanding of the knowledge, attitudes, and behavior patterns of doctors is essential for developing successful strategies to improve lung cancer screening. This study aims to identify the attitudes, beliefs, referral practices, and knowledge of lung cancer screening among physicians in Saudi Arabia.

Methods: An online survey was conducted from July to December 2023 to investigate the attitudes, beliefs, referral practices, and knowledge of lung cancer screening, and adherence to lung cancer screening recommendations among physicians in Saudi Arabia. Internal medicine, family medicine, and pulmonology physicians of all levels (consultants, senior registrars, and residents) who are currently practicing medicine in Saudi Arabia formed the study population. This study employed a previously developed questionnaire. Binary logistic regression analysis was employed to identify factors that indicate a better degree of knowledge and a positive attitude toward lung cancer screening.

Results: This study involved a total of 96 physicians. The study participants demonstrated a significant degree of understanding regarding lung cancer screening, with an average knowledge score of 5.8 (SD: 1.7) out of 8, equivalent to 72.5% of the highest possible score. The accuracy rate for knowledge items varied from 44.8% to 91.7%. The study participants had a moderately favorable attitude toward lung cancer screening, as shown by a mean attitude score of 14.4 (SD: 3.7) out of a maximum possible score of 30, which corresponds to 48.0% of the highest achievable score. Around 36.5% of the survey participants reported engaging in the practice of discussing the results of lung cancer screening with patients. The primary obstacles frequently cited were challenges in patient scheduling, insufficient time to discuss lung cancer screening during clinic appointments, and patient refusal, constituting 59.4%, 53.1%, and 53.1% of the identified barriers, respectively. Physicians in Saudi Arabia, particularly those employed in private hospitals, demonstrated a higher level of knowledge of lung cancer screening compared to others (p < 0.05). In contrast, individuals with 11-15 years of experience were shown to have a 78.0% lower likelihood of being educated about lung cancer screening compared to their counterparts (p < 0.05).

Conclusion: The study's results indicate that there is a need for the development of specialized educational initiatives aimed at Saudi Arabian physicians, particularly those with 11 to 15 years of experience who exhibit a limited understanding of lung cancer screening. Utilizing programs that provide continuing medical education would aid in their education. There is a need to facilitate communication between physicians and patients. It is critical to address the identified issues, such as streamlining the appointment scheduling process and ensuring patients have sufficient time during clinic visits. Furthermore, it is critical for the success of nationwide screening initiatives to foster collaboration between the public and private healthcare sectors.

## Introduction

Lung cancer remains the primary cause of death connected to cancer on a worldwide scale. In 2015, around 20% of deaths connected to cancer were attributed to lung cancer, and tobacco usage was responsible for 25% of these fatalities [1]. This disease is often diagnosed at an advanced stage due to its early asymptomatic progression, leading to a poor prognosis at the time of diagnosis [2]. Timely and accurate diagnosis is essential since the prognosis depends on the stage of detection: 59.8% of patients with localized cancer survive for more than five years, whereas only 6.3% survive when the cancer has metastasized to other parts of the body. Unfortunately, only 18% of lung cancer cases are detected at an early stage, while 56% are identified after the cancer has spread to other areas of the body [3]. The prevalence of lung cancer in Saudi Arabia has risen by over 3.5-fold during the past three to four decades [4]. Based on statistics from the Saudi Cancer Registry, lung cancer was the sixth most common cancer among Saudi males and the 12th most common disease among Saudi females in 2018. Saudi Arabia documented 504 new cases of lung cancer, representing 3.2% of the total number of recently identified cases.

In a recent study, the prevalence rate of lung cancer was found to be 69.2% among males and 30.8% among females [5]. By conducting screening, we can detect this potentially fatal disease in individuals who have a higher susceptibility at an early phase, allowing us to provide more effective medical intervention. Obtaining a deep understanding of the knowledge, attitudes, and behavior patterns of doctors is essential for developing successful strategies to improve lung cancer screening [6]. Several methods have been proposed to improve the chances of survival, including chest radiography (X-ray), sputum cytology, low-dose computed tomography (LDCT), and molecular biomarkers [7]. Research has shown that LDCT scans significantly reduce the mortality rate associated with lung cancer [8]. The National Lung Screening Trial conducted a comparative study in 2011, examining the effectiveness of LDCT screening vs. chest radiograph (CXR) screening in detecting lung cancer. The findings demonstrated a noteworthy decrease of 20% in lung cancer mortality and a significant decrease of 6.7% in total mortality [9]. In March 2013, the United States Preventive Services Task Force (USPSTF) recommended that individuals aged 55 to 80 years, who have a smoking history of 30 packs of cigarettes per year and are currently smoking or have quit within the past 15 years, should have an annual LDCT screening, regardless of whether they exhibit any symptoms [10]. The American Cancer Society (ACS) recommends that individuals aged 55 to 74 years, who are in good general health, have a smoking history comparable to 30 packs per year, and are current smokers or have quit within the past 15 years, should have LDCT screening [11]. The USPSTF recommendation was updated in March 2021 to lower the minimum age to 50 and the pack-year exposure to 20 [12]. In Saudi Arabia, the guidelines for lung cancer screening recommend screening individuals aged 55 to 77 years who fulfill specific criteria. These criteria include having a smoking history of at least 30 years, being a current smoker, being a former smoker who quit within the last 15 years, or not having undergone a chest CT scan in the previous year. In addition, they strongly advise against the utilization of LDCT screening in patients with severe liver disease, chronic obstructive pulmonary disease (COPD) accompanied by hypoventilation and hypoxia, and New York Heart Association (NYHA) class IV heart failure [13]. Although LDCT screening has been acknowledged for over 10 years as a successful approach to reducing lung cancer fatalities and has been recommended for over six years, its utilization remains disappointingly low. Only 19.2% of eligible patients underwent this screening in 2018. This study aims to identify the attitudes, beliefs, referral practices, and knowledge of lung cancer screening, and adherence to lung cancer screening recommendations among internal medicine, family medicine, and pulmonology physicians in Saudi Arabia.

## Materials and methods

Study design

An online survey was conducted from July to December 2023 to investigate the attitudes, beliefs, referral practices, and knowledge of lung cancer screening, and adherence to lung cancer screening recommendations among physicians in Saudi Arabia.

Study population

Internal medicine, family medicine, and pulmonology physicians of all levels (consultants, senior registrars, and residents) who are currently practicing medicine in Saudi Arabia formed the study population. There was no restriction based on the gender or the demographic characteristics of the study participants. Any physician with a different specialty or who does not currently practice medicine in Saudi Arabia was excluded.

Sampling technique

This study utilized a convenient sampling strategy. The survey link was disseminated to the intended research participants in Saudi Arabia using several social media platforms (Facebook, WhatsApp, Twitter, Snapchat, and Instagram) using a Google Forms link (Google, Mountain View, CA). Additionally, a cover letter was included along with a consent form. Participation was optional, allowing anyone to withdraw at their discretion. All responses were provided anonymously, without any monitoring of email addresses or any identifiable data.

Questionnaire tool

This study employed a previously developed questionnaire that was produced by Alrabiah et al. [14]. The original questionnaire was constructed based on the recommendations and existing literature [14]. The study questionnaire comprised five sections. The first section was devised to gather information about the physician's demographic characteristics, encompassing eight factors: age, gender, nationality, specialty, level of experience, years of experience, region of practice, and site of practice. The second section comprised eight items that examined physicians' understanding of LDCT lung screening. The third section included five items that evaluated the participants' attitudes toward LDCT lung screening, while the fourth section consisted of five items that investigated the physicians' practices of LDCT lung screening. Lastly, the fifth section comprised 11 elements that represented the challenges encountered in LDCT lung screening.

Questionnaire piloting

During the pilot phase, the questionnaire was randomly disseminated to various regions of Saudi Arabia among a sample of 20 participants to assess the internal consistency of the study. These participants did not make any contributions to the findings of the primary investigation. The measurement of the reliability of the attitude and practices sections of the questionnaire was conducted. The Cronbach's alpha score was used to assess the overall reliability of the attitude and practices sections. The measure yielded values of 0.785 and 0.771 for the attitude and practices sections, respectively, indicating an acceptable level of internal consistency.

Ethical approval

The ethical approval for this study was obtained from the ethical research committee of the Institutional Review Board (IRB) of Imam Mohammad Ibn Saud Islamic University (IMSIU), Riyadh, Saudi Arabia, wherein they reviewed and approved this project (HAPO-01-R-0011; Project No.: 517/2023). Informed consent was obtained from all participants.

Data analysis

The data were analyzed using the Statistical Package for Social Science (SPSS) software version 28 (IBM Corp., Armonk, NY). Categorical variables were represented using numerical values and percentages (%), whereas continuous variables were displayed as the mean and standard deviation (SD). The normality assessment was conducted utilizing the Shapiro-Wilk test and Kolmogorov-Smirnov test. The study employed binary logistic regression analysis to identify factors that indicate a better degree of knowledge and a positive attitude toward lung cancer screening. The binary logistic regression analysis employed dummy variables, which were determined based on the mean knowledge and attitude score of the study sample, serving as the threshold values. The statistical significance level was set at 5.0%.

## Results

Participants' demographic and practice characteristics

A total of 96 physicians were involved in this study. More than half of the study participants (61.5%; n = 59) were aged 25-34 years and were males (53.1%; n = 51). The vast majority of the study participants (89.6%; n = 86) were Saudis. Around 58.3% (n = 56) of the study participants specialized in family medicine. Around 45.8% (n = 44) of the study participants were residents. More than half of the study participants (61.5%; n = 59) reported that they have an experience of less than five years. Of the study participants, 50% (n = 48) reported that they practice their profession in the central region of Saudi Arabia. Around 28.1% (n = 27) of the study participants reported that they work at private hospitals. For further details on participants' demographic and practice characteristics, refer to Table 1.

**Table 1:** Participants' demographic and practice characteristics.

Variable	Frequency	Percentage
Age		
25-34 years	59	61.5%
35-44 years	21	21.9%
45-54 years	9	9.4%
55-64 years	4	4.2%
65 years and older	3	3.1%
Gender		
Males	51	53.1%
Nationality		
Saudi	86	89.6%
Specialty		
Internal medicine	27	28.1%
Family medicine	56	58.3%
Pulmonologist	6	6.3%
Doctor of Medicine (MD)	7	7.3%
Level of experience		
Consultant	25	26.0%
Fellow	2	2.1%
Senior registrar	15	15.6%
Registrar	10	10.4%
Resident	44	45.8%
Years of experience		
Less than 5 years	59	61.5%
5-10 years	15	15.6%
11-15 years	10	10.4%
16-20 years	5	5.2%
More than 20 years	7	7.3%
Region of practice		
Central region	48	50.0%
Eastern region	22	22.9%
Western region	13	13.5%
Northern region	4	4.2%
Southern region	9	9.4%
Place of practice		
Primary healthcare center	27	28.1%
Medical city/specialized hospital	21	21.9%
Military, security, or national guard hospital	14	14.6%
University hospital	13	13.5%
Private hospital	11	11.5%
Ministry of Health secondary hospital	10	10.4%

Knowledge of lung cancer screening

Table 2 below presents the percentage of right responses to knowledge of lung cancer screening items. The study participants showed a high level of knowledge of lung cancer screening with a mean knowledge score of 5.8 (SD: 1.7) out of 8, which represents 72.5% of the maximum attainable score. The percentage of right responses for knowledge items ranged between 44.8% (n = 43) and 91.7% (n = 88). The most commonly answered question correctly was identifying that “current smoker or former smoker within last 15 years” is a smoking history that makes a patient eligible for LDCT lung cancer screening. The least commonly answered question correctly was identifying that LDCT lung cancer screening should be performed annually.

**Table 2:** Percentage of right responses to knowledge of lung cancer screening items. LDCT: low-dose computed tomography.

Knowledge item	Frequency of correct answers	Percentage
What is the smoking history that makes a patient eligible for LDCT lung cancer screening? “Current smoker or former smoker within last 15 years”	88	91.7%
Lung cancer screening reduces cancer mortality. “Agree”	84	87.5%
Low-dose computed tomography (LDCT) is better than X-ray in lung cancer screening. “Agree”	84	87.5%
What is the presentation of a patient who is eligible for LDCT lung cancer screening? “Asymptomatic and in fairly good health”	72	75.0%
The age of the patient eligible for LDCT lung cancer screening. “Between 50 and 80 years”	65	67.7%
What is the presentation of a patient who is eligible for LDCT lung cancer screening? “Has at least a 20 pack-year of smoking”	63	65.6%
Lung cancer screening is cost-effective. “Agree”	59	61.5%
The frequency of LDCT lung cancer screening. “Annually”	43	44.8%

Attitude toward lung cancer screening

The study participants showed moderately positive attitudes toward lung cancer screening with a mean attitude score of 14.4 (SD: 3.7) out of 30, which represents 48.0% of the maximum attainable score. The most commonly agreed upon statement was that “they believe that the side effects of lung cancer treatment (surgery, chemotherapy, and radiotherapy) outweigh the survival benefit” and that "lung cancer screening will place a burden on the healthcare system” with 40.7% (n = 39). The least commonly agreed upon statement was that “they are convinced that screening for lung cancer is beneficial for patients” with 6.2% (n = 6). For further details on participants’ responses to attitude items, refer to Table 3.

**Table 3:** Attitude toward lung cancer screening.

Attitude item	Strongly disagree	Disagree	Neutral	Agree	Strongly agree
I am convinced that screening for lung cancer is beneficial for patients.	64.6%	14.6%	14.6%	3.1%	3.1%
I have enough knowledge to explain the advantages and disadvantages of lung cancer screening to my patients.	30.2%	31.3%	26.0%	9.4%	3.1%
I believe lung cancer screening will place a burden on the healthcare system.	19.8%	13.5%	26.0%	18.8%	21.9%
I believe that the side effects of lung cancer treatment (surgery, chemotherapy, and radiotherapy) outweigh the survival benefit.	13.5%	26.0%	19.8%	18.8%	21.9%
I believe that lung cancer screening can improve the patient's management options.	62.5%	22.9%	6.3%	4.2%	4.2%

Practices related to lung cancer screening

Table 4 presents participants’ practices related to lung cancer screening. The most commonly reported practice among the study participants related to lung cancer screening was that they “discuss the results of lung cancer screening with patients” with 36.5% (n = 35). The least commonly reported practice among the study participants related to lung cancer screening was that they “order chest X-ray for lung cancer screening” with 8.3% (n = 8).

**Table 4:** Practices related to lung cancer screening. LDCT: low-dose computed tomography.

Practice item	Never	Rarely	Sometimes	Always
Answer these questions based on your previous practices with your patients over the past 12 months.				
I discuss the benefits and risks of lung cancer screening with eligible patients.	14.6%	21.9%	31.3%	32.3%
I order LDCT for lung cancer screening.	35.4%	17.7%	22.9%	24.0%
I order a chest X-ray for lung cancer screening.	47.9%	20.8%	22.9%	8.3%
I refer patients to a lung cancer screening program.	33.3%	24.0%	19.8%	22.9%
I discuss the results of lung cancer screening with patients.	26.0%	19.8%	17.7%	36.5%

Barriers related to lung cancer screening

Table 5 presents barriers related to lung cancer screening reported by the study participants. The most commonly reported barriers were having difficulties in scheduling patients, not having enough time to address lung cancer screening during a clinic visit, and patient refusal accounting for 59.4% (n = 57), 53.1% (n = 51), and 53.1% (n = 51), respectively.

**Table 5:** Barriers related to lung cancer screening.

Variable	Frequency	Percentage
Difficult scheduling patients	57	59.4%
Not enough time to address lung cancer screening during a clinic visit	51	53.1%
Patient refusal	51	53.1%
Concerns of the patients about radiation exposure	49	51.0%
Fear of the patients from other incidental findings	48	50.0%
Limited/lack of CT machines	43	44.8%
Cost of screening	38	39.6%
Consequences of false positive results	36	37.5%
Leadership at practice is not supportive of lung cancer screening	29	30.2%
Insufficient evidence to warrant a screening program	27	28.1%
Inconsistent recommendations about lung cancer screening	23	24.0%

Predictors of participants’ knowledge and positive attitude

Table 6 below presents predictors of participants’ knowledge and positive attitudes. Binary logistic regression analysis identified that Saudi physicians and those who work at private hospitals were more likely to be knowledgeable about lung cancer screening compared to others (p < 0.05). On the other hand, those who have an experience of 11-15 years were 78.0% less likely to be knowledgeable about lung cancer screening compared to others (p < 0.05). Binary logistic regression analysis did not identify any significant difference in physicians’ attitudes toward lung cancer screening (p > 0.05).

**Table 6:** Predictors of participants’ knowledge and positive attitude * P < 0.05.

Variable	Odds ratio of being knowledgeable (95% CI)	Odds ratio of having a positive attitude (95% CI)
Age		
25-34 years	1.00	
35-44 years	3.90 (0.33-45.66)	-
45-54 years	3.25 (0.25-41.91)	3.10 (0.31-31.58)
55-64 years	1.60 (0.10-24.70)	3.30 (0.29-37.10)
65 years and older	2.00 (0.09-44.35)	2.40 (0.18-32.88)
Gender		
Female	1.00	
Male	0.66 (0.29-1.51)	1.39 (0.62-3.11)
Nationality		
Non-Saudi	1.00	
Saudi	4.4 (1.05-18.08)*	0.41 (0.10-1.69)
Specialty		
Internal medicine	1.00	
Family medicine	1.69 (0.66-4.34)	0.40 (0.16-1.05)
Pulmonologist	4.00 (0.41-39.0)	0.25 (0.04-1.63)
Doctor of Medicine (MD)	0.13 (0.01-1.26)	0.67 (0.12-3.64)
Level of experience		
Consultant	1.00	
Fellow	-	-
Senior registrar	1.55 (0.38-6.31)	1.63 (0.44-5.95)
Registrar	0.84 (0.19-3.80)	1.63 (0.37-7.20)
Resident	0.81 (0.30-2.24)	1.08 (0.41-2.89)
Years of experience		
Less than 5 years	1.00	
5-10 years	1.03 (0.31-3.41)	0.85 (0.27-2.63)
11-15 years	0.22 (0.05-0.94)*	3.87 (0.76-19.76)
16-20 years	0.34 (0.05-2.22)	0.64 (0.10-4.14)
More than 20 years	1.28 (0.23-7.30)	0.39 (0.07-2.15)
Region of Practice		
Central region	1.00	
Eastern region	1.05 (0.37-2.99)	0.54 (0.19-1.50)
Western region	2.00 (0.49-8.24)	0.91 (0.27-3.11)
Northern region	0.60 (0.08-4.64)	2.33 (0.23-24.08)
Southern region	0.30 (0.07-1.35)	0.39 (0.09-1.74)
Place of practice		
Primary healthcare center	1.00	
Medical city/specialized hospital	2.40 (0.60-9.67)	1.54 (0.36-6.60)
Military, security, or national guard hospital	3.00 (0.70-12.93)	0.99 (0.24-4.09)
University hospital	1.33 (0.29-6.04)	0.41 (0.09-1.92)
Private hospital	4.67 (1.33-16.34)*	0.42 (0.13-1.36)
Ministry of Health secondary hospital	1.60 (0.37-0.95)	0.23 (0.05-1.14)

## Discussion

Lung cancer is a prevalent and deadly disease that is strongly linked to long-term tobacco smoking [15,16]. Saudi Arabia has established guidelines for screening and treating lung cancer, which cover both primary and secondary prevention of the disease. These guidelines include recommendations for screening high-risk individuals [13,17]. Recent advancements in our understanding of the biology of the disease, the use of predictive biomarkers, and improvements in treatment have resulted in significant progress over the past two decades [18]. Therefore, this study aimed to evaluate the knowledge, attitudes, and practices of Saudi physicians regarding lung cancer screening. It also aimed to assess their adherence to the recommended screening guidelines.

The study participants demonstrated a high level of understanding of lung cancer screening, as evidenced by an average knowledge score of 5.8 out of 8 (SD: 1.7). This score corresponds to 72.5% of the highest possible attainable score. These results are similar to those observed among primary care physicians in Chicago, USA, where 72% of them possessed adequate knowledge about lung cancer screening [19]. However, in France, primary care physicians, including pulmonologists, lack sufficient knowledge or understanding of lung cancer screening [20]. It is worth noting that early detection of lung cancer through screening can significantly improve patient outcomes, especially when curative surgical intervention is possible [21]. In Saudi Arabia, a team of experts from different fields has developed guidelines for preventing and detecting lung cancer. These guidelines emphasize the important role of healthcare professionals, especially physicians, in implementing measures to control tobacco use and promoting early diagnosis. This is particularly important because there is a high number of lung cancer patients who are smokers in Saudi Arabia. Therefore, in addition to campaigns to help people quit smoking, it is crucial to have effective screening programs in primary care settings. This will enable early diagnosis and referral to specialists, and improve patient outcomes.

The study findings revealed that the question with the highest rate of right answers was the recognition of "current smoker or former smoker within the past 15 years" as a smoking history that qualifies a patient for LDCT lung cancer screening. On the other hand, it is important to note that LDCT has been proven to be an effective method for screening lung cancer. It has the potential to reduce mortality by 20%. Therefore, primary care providers should be aware of the resources available in their communities for both LDCT lung cancer screening and smoking cessation. LDCT screening is particularly crucial for detecting lung cancers at an early stage, especially in high-risk patients. Studies have shown that LDCT screening can significantly reduce lung cancer mortality in populations with a history of significant tobacco exposure. In addition, annual lung cancer screening using LDCT can decrease lung cancer mortality by 20% compared to chest X-rays for individuals who are current or former heavy smokers [22]. According to the China National Lung Cancer Screening guideline, it is recommended that individuals aged 50-74 years, who have a smoking history of at least 20 pack-years and currently smoke or have quit within the past five years, undergo annual lung cancer screening with LDCT [23].

The study participants displayed a moderately positive attitude toward lung cancer screening, as evidenced by an average attitude score of 14.4 out of 30 (SD: 3.7), which corresponds to 48.0% of the highest possible score. The prevailing consensus was that the adverse consequences of lung cancer treatment (including surgery, chemotherapy, and radiotherapy) are more significant than the potential for increased survival. Additionally, there is a widespread sense that lung cancer screening will impose a strain on the healthcare system, with 40.7% of individuals holding this view. On the other hand, the statement that received the least agreement was the belief that screening for lung cancer is beneficial for patients, with only 6.2% of respondents agreeing. This discrepancy may be explained by the fact that physicians' attitudes toward lung cancer screening vary depending on their specialty. Pulmonologists are more likely to view it as beneficial and cost-effective [24]. Additionally, it was found that family physicians generally have positive attitudes toward LDCT lung cancer screening, but may lack knowledge about its use [25]. However, there are barriers that physicians face when it comes to lung cancer screening. These barriers include the complexity of the screening process and the challenge of allocating limited time and resources to integrate the necessary components for lung cancer screening [26]. Furthermore, there is a belief that the introduction of lung cancer screening, specifically using LDCT, could impose a substantial financial strain on the healthcare system. The cost-effectiveness of lung cancer screening is still uncertain, primarily because of the expensive nature of lung cancer treatment, which includes hospital stays, outpatient appointments, and medication expenses. Consequently, despite the important role of lung cancer screening in identifying and reducing the death rate from lung cancer, its effectiveness in specific situations is still a topic of debate.

The most frequently reported practice among the study participants was engaging in discussions with patients about the results of lung cancer screening, with a prevalence of 36.5%. It was observed that individuals who were potentially eligible for lung cancer screening were more inclined to have these discussions with their healthcare providers [27]. In fact, a centralized counseling approach and shared decision-making between physicians and patients had a significant impact on patients' understanding of the eligibility criteria, benefits, and risks associated with lung cancer screening, particularly with LDCT [28]. Conversely, the study participants rarely reported the practice of ordering chest X-rays for lung cancer screening, with only 8.3% doing so. It has been found that chest X-ray screening is not effective in detecting lung cancer at an early stage [29]. In contrast, LDCT has been shown to be a more effective screening tool for lung cancer, with higher rates of positive results and a greater ability to detect cancer in its early stages [30,31]. However, concerns have been raised about the high number of false-positive results and the potential for overdiagnosis. This indicates a need for further research and improvement in screening methods [32].

The most frequently mentioned barriers included difficulties in scheduling patients, insufficient time to address lung cancer screening during clinic visits, and patient refusal, accounting for 59.4%, 53.1%, and 53.1%, respectively. Patient appointment scheduling delays were identified as a significant contributing factor to the common occurrence of delays in diagnosing and treating lung cancer [33]. Additionally, it was reported that time constraints during patient visits pose numerous challenges for clinicians in implementing lung cancer screening [34]. Furthermore, patient refusal of lung cancer screening and treatment is a complex issue influenced by various factors, such as fear, inconvenience, and perceived low susceptibility. These reasons contribute to the decline in participation in lung cancer screening [35]. Furthermore, the expense of the test and the extent of insurance coverage [36], in conjunction with individual and health-system level factors such as sociodemographic characteristics, lack of awareness, and skepticism of the medical system [37], can also serve as additional obstacles for lung cancer screening.

Physicians in Saudi Arabia, particularly those employed in private institutions, exhibit a greater probability of obtaining knowledge regarding lung cancer screening in comparison to their counterparts (p < 0.05). In contrast, persons who had 11-15 years of experience were shown to have a 78.0% lower likelihood of possessing knowledge about lung cancer screening compared to others (p < 0.05). In Saudi Arabia, a team of experts from different fields has created guidelines for preventing and detecting lung cancer [13]. These guidelines are crucial because lung cancer has high mortality rates and is often diagnosed at a late stage in the country [38]. However, there is still ongoing discussion about whether to implement a national screening program [13]. Additionally, the experience and practices of physicians in lung cancer screening are influenced by various factors [39]. Research has shown that while there is interest in screening, knowledge and practices differ among different groups of physicians [20].

The study findings suggest that it would be beneficial to prioritize specific educational programs for healthcare workers in Saudi Arabia, namely, those with 11-15 years of experience who had a limited understanding of lung cancer screening. Continuous medical education initiatives should be utilized to augment their comprehension. Furthermore, it is imperative to promote and support transparent dialogue between healthcare professionals and patients, considering the favorable disposition toward communicating screening outcomes. It is crucial to overcome obstacles that have been identified, such as improving the efficiency of appointment procedures and ensuring that enough time is allocated for clinic appointments. Moreover, it is essential to foster cooperation between the public and private healthcare sectors to effectively execute national screening programs, given the increased probability that private hospital staff hold expertise in lung cancer screening. The purpose of these recommendations is to enhance the effectiveness of lung cancer screening procedures and enhance patient outcomes in Saudi Arabia.

Study limitations

This study has limitations. The cross-sectional survey study design restricted our ability to examine causality among the study variables. Therefore, we were not able to follow up with the study participants and examine the change in their outcomes. The online survey might have affected the generalizability of the study findings, as we might have missed participants who do not use social media platforms. Selection bias and recall bias are another two possible biases in this study. This is an exploratory survey study with no sample size calculation; therefore, our findings should be interpreted carefully.

## Conclusions

Based on the findings of the study, it is suggested that targeted educational programs be made for physicians in Saudi Arabia. The most commonly reported barriers were having difficulties in scheduling patients, not having enough time to address lung cancer screening during a clinic visit, and patient refusal accounting. To help them learn more, they should use programs that offer ongoing medical education. Efforts should be made to make it easier for physicians and patients to talk to each other. It is important to get rid of the problems that have been found, like making it easier to make appointments and giving patients enough time during clinic trips. Also, encouraging collaboration between the public and private healthcare sectors is important for the success of national screening programs.

## References

[1] Kocarnik JM, Compton K, Dean FE (2022). Cancer incidence, mortality, years of life lost, years lived with disability, and disability-adjusted life years for 29 cancer groups from 2010 to 2019: a systematic analysis for the Global Burden of Disease Study 2019. JAMA Oncol.

[2] Goldstraw P, Crowley J, Chansky K (2007). The IASLC Lung Cancer Staging Project: proposals for the revision of the TNM stage groupings in the forthcoming (seventh) edition of the TNM Classification of Malignant Tumours. J Thorac Oncol.

[3] (2023). SEER Cancer Statistics Review (CSR) 1975-2018. https://seer.cancer.gov/archive/csr/1975_2018/index.html.

[4] Althubiti MA, Nour Eldein MM (2018). Trends in the incidence and mortality of cancer in Saudi Arabia. Saudi Med J.

[5] Saudi Health Council (2023). Cancer incidence report in Kingdom of Saudi Arabia 2018. https://shc.gov.sa/Arabic/NCC/Activities/AnnualReports/2018.pdf.

[6] Carter-Bawa L, Walsh LE, Schofield E, Williamson TJ, Hamann HA, Ostroff JS (2023). Lung cancer screening knowledge, attitudes, and practice patterns among primary and pulmonary care clinicians. Nurs Res.

[7] Gouvinhas C, De Mello RA, Oliveira D (2018). Lung cancer: a brief review of epidemiology and screening. Future Oncol.

[8] Huang KL, Wang SY, Lu WC, Chang YH, Su J, Lu YT (2019). Effects of low-dose computed tomography on lung cancer screening: a systematic review, meta-analysis, and trial sequential analysis. BMC Pulm Med.

[9] Aberle DR, Adams AM, Berg CD (2011). Reduced lung-cancer mortality with low-dose computed tomographic screening. N Engl J Med.

[10] Moyer VA (2014). Screening for lung cancer: U.S. Preventive Services Task Force recommendation statement. Ann Intern Med.

[11] Siegel RL, Miller KD, Fuchs HE, Jemal A (2021). Cancer statistics, 2021. CA Cancer J Clin.

[12] Krist AH, Davidson KW, Mangione CM (2021). Screening for lung cancer: US Preventive Services Task Force recommendation statement. JAMA.

[13] Jazieh AR, AlGhamdi M, AlGhanem S (2018). Saudi lung cancer prevention and screening guidelines. Ann Thorac Med.

[14] Alrabiah B, Alshammari S, Alwatban O (2021). Knowledge, attitude, and behavior of family medicine residents regarding low-dose computed-tomography lung cancer screening at primary care setting in Riyadh, Saudi Arabia in 2020. Med Sci.

[15] Brody H (2020). Lung cancer. Nature.

[16] Hoffman PC, Mauer AM, Vokes EE (2000). Lung cancer. Lancet.

[17] Howington J (2018). Saudi lung cancer prevention and screening guidelines. Ann Thorac Med.

[18] Thai AA, Solomon BJ, Sequist LV, Gainor JF, Heist RS (2021). Lung cancer. Lancet.

[19] Mukthinuthalapati VV, Putta A, Farooq MZ, Singh SR, Gupta S, Smith S (2020). Knowledge, attitudes, and practices pertaining to lung cancer screening among primary care physicians in a public urban health network. Clin Lung Cancer.

[20] Couraud S, Girard N, Erpeldinger S, Gueyffier F, Devouassoux G, Llorca G, Souquet PJ (2013). Physicians' knowledge and practice of lung cancer screening: a cross-sectional survey comparing general practitioners, thoracic oncologists, and pulmonologists in France. Clin Lung Cancer.

[21] Gomez M, Silvestri GA (2008). Lung cancer screening. Am J Med Sci.

[22] Karachaliou N, Sosa AE, Rosell R (2016). Annual or biennial lung cancer CT screening?. J Thorac Dis.

[23] Zhou Q, Fan Y, Wang Y (2018). China national lung cancer screening guideline with low-dose computed tomography (2018 version). Zhongguo Fei Ai Za Zhi.

[24] Henderson LM, Marsh MW, Benefield TS (2019). Opinions and practices of lung cancer screening by physician specialty. N C Med J.

[25] Ersek JL, Eberth JM, McDonnell KK, Strayer SM, Sercy E, Cartmell KB, Friedman DB (2016). Knowledge of, attitudes toward, and use of low-dose computed tomography for lung cancer screening among family physicians. Cancer.

[26] Henderson LM, Benefield TS, Bearden SC (2019). Changes in physician knowledge, attitudes, beliefs, and practices regarding lung cancer screening. Ann Am Thorac Soc.

[27] Chalian H, Khoshpouri P, Iranmanesh AM, Mammarappallil JG, Assari S (2019). Lung cancer screening patient-provider discussion: where do we stand and what are the associated factors?. SAGE Open Med.

[28] Mazzone PJ, Tenenbaum A, Seeley M, Petersen H, Lyon C, Han X, Wang XF (2017). Impact of a lung cancer screening counseling and shared decision-making visit. Chest.

[29] Gavelli G, Giampalma E (2000). Sensitivity and specificity of chest X-ray screening for lung cancer: review article. Cancer.

[30] Gohagan JK, Marcus PM, Fagerstrom RM (2005). Final results of the Lung Screening Study, a randomized feasibility study of spiral CT versus chest X-ray screening for lung cancer. Lung Cancer.

[31] Murugan VA, Kalra MK, Rehani M, Digumarthy SR (2015). Lung cancer screening: computed tomography radiation and protocols. J Thorac Imaging.

[32] van der Aalst CM, Ten Haaf K, de Koning HJ (2016). Lung cancer screening: latest developments and unanswered questions. Lancet Respir Med.

[33] Lin Y, Qureshi M, Tapan U (2021). Reducing delays to lung cancer treatment through systematic consult scheduling: a multidisciplinary quality improvement initiative at a safety-net hospital. J Clin Oncol.

[34] Melzer AC, Golden SE, Ono SS, Datta S, Triplette M, Slatore CG (2020). “We just never have enough time”: clinician views of lung cancer screening processes and implementation. Ann Am Thorac Soc.

[35] Patel D, Akporobaro A, Chinyanganya N, Hackshaw A, Seale C, Spiro SG, Griffiths C (2012). Attitudes to participation in a lung cancer screening trial: a qualitative study. Thorax.

[36] Delmerico J, Hyland A, Celestino P, Reid M, Cummings KM (2014). Patient willingness and barriers to receiving a CT scan for lung cancer screening. Lung Cancer.

[37] Lei F, Lee E (2019). Barriers to lung cancer screening with low-dose computed tomography. Oncol Nurs Forum.

[38] Alghamdi HI, Alshehri AF, Farhat GN (2018). An overview of mortality & predictors of small-cell and non-small cell lung cancer among Saudi patients. J Epidemiol Glob Health.

[39] Henderson S, DeGroff A, Richards TB, Kish-Doto J, Soloe C, Heminger C, Rohan E (2011). A qualitative analysis of lung cancer screening practices by primary care physicians. J Community Health.

